# Reduced inhibition control ability in children with ADHD due to coexisting learning disorders: an fNIRS study

**DOI:** 10.3389/fpsyt.2024.1326341

**Published:** 2024-05-08

**Authors:** Fulin Liu, Xia Chi, Dongchuan Yu

**Affiliations:** ^1^ Key Laboratory of Child Development and Learning Science of Ministry of Education, School of Biological Science and Medical Engineering, Southeast University, Nanjing, China; ^2^ Department of Child Health Care, Women’s Hospital of Nanjing Medical University, Nanjing Maternity and Child Health Care Hospital, Nanjing, Jiangsu, China; ^3^ Henan Provincial Medical Key Lab of Child Developmental Behavior and Learning, The Third Affiliated Hospital of Zhengzhou University, Zhengzhou, Henan, China

**Keywords:** attention deficit/hyperactivity disorder, learning disabilities, comorbidity, functional near infrared spectroscopy, two-choice Oddball paradigm, inhibition control

## Abstract

**Introduction:**

Inhibition control, as the core component of executive function, might play a crucial role in the understanding of attention deficit/hyperactivity disorder (ADHD) and specific learning disorders (SLD). Inhibition control deficits have been observed in children with ADHD or SLD. This study sought to test in a multi-modal fashion (i.e., behavior and plus brain imaging) whether inhibition control abilities would be further deteriorated in the ADHD children due to the comorbidity of SLD.

**Method:**

A total number of 90 children (aged 6-12 years) were recruited, including 30 ADHD, 30 ADHD+SLD (children with the comorbidity of ADHD and SLD), and 30 typically developing (TD) children. For each participant, a 44-channel functional near infrared spectroscopy (fNIRS) equipment was first adopted to capture behavioral and cortical hemodynamic responses during a two-choice Oddball task (a relatively new inhibition control paradigm). Then, 50 metrics were extracted, including 6 behavioral metrics (i.e., OddballACC, baselineACC, totalACC, OddballRT, baselineRT, and totalRT) and 44 beta values in 44 channels based on general linear model. Finally, differences in those 50 metrics among the TD, ADHD, and ADHD+SLD children were analyzed.

**Results:**

Findings showed that: (1) OddballACC (i.e., the response accuracy in deviant stimuli) is the most sensitive metric in identifying the differences between the ADHD and ADHD+SLD children; and (2) The ADHD+SLD children exhibited decreased behavioral response accuracy and brain activation level in some channels (e.g., channel CH35) than both the ADHD and TD children.

**Discussion:**

Findings seem to support that inhibition control abilities would be further decreased in the ADHD children due to the comorbidity of SLD.

## 1 Introduction

Attention-deficit/hyperactivity disorder (ADHD) and specific learning disorders (SLDs) have been the most common mental disorders in childhood (1). The prevalence of ADHD in children is about 5%, while that of SLD is about 2-8% (2–7). There is significant comorbidity between the two illnesses (2). Twenty to seventy percent of children with a clinical diagnosis of ADHD also suffer from SLD, while 20% to 28% of children with SLD also exhibit clinically significant symptoms of ADHD (2–7). The current study aims to examine similarities and differences between cognitive and neural features of children with ADHD, children with ADHD+SLD (the comorbidity of ADHD and SLD), and a control group of healthy children to find out whether specific and/or shared deficits can be identified for the groups.

Executive functioning (EF) refers to the psychological processes that maintain an appropriate problem-solving set to attain a future goal (8, 9). EF involves multiple dispersed neural networks, including the prefrontal cortex, thalamus, and basal ganglia (10). Children with different EF profiles may have differences in academic achievement and mental health outcomes. The gender and age may play important roles in the development of EF across the lifespan (11). Even though EF may be adopted to map the structure of neurodevelopmental conditions (including ADHD and SLD), but evidence for associations between specific EF profiles and specific neurodevelopmental conditions is mixed (10–13).

It has been well documented that EF deficits have been revealed in ADHD and SLD (10, 14–18). For instance, a meta-analysis of 83 studies (10) identified 13 EF measures that were frequently administered in previous ADHD studies, and found that the strongest and most consistent effects were obtained on measures of inhibition control, vigilance, working memory, and planning. While, children with SLD usually exhibit poor EF, especially observed in inhibition control (14, 18) and working memory (15, 16).

Additionally, inhibition control is the core component of EF, and may play a crucial role in academic performances and achievements (17–25), which are significantly related with SLD. On the other hand, impulsivity, which involves the process of inhibition control, is assumed to be a major characteristic of ADHD (17). Hence, the ADHD or SLD children might exhibit deficits in inhibition control (17). The main motivation of the current study is to understand inhibitory control deficits in the comorbidity of ADHD and SLD in a multi-modal fashion (i.e., behavior plus brain imaging).

The Go/NoGo (GNG) and Stop-signal (SS) paradigms have been extensively employed in prior inhibitory control research, yet both have some technical flaws to some extent (20). In a GNG task, participants only need to respond to “Go” trials, rather than “NoGo” trials; “Go” trials require motor response, while “NoGo” trails do not. These differences between “Go” and “NoGo” trials result in that the GNG paradigm cannot provide effective behavioral metrics of response inhibition (20, 21). While, in a SS task, participants are required to respond quickly to all “GO” trails, but have to stop response when “Stop” trails present randomly. This random presence of “Stop” trails leads to that both behavioral and physiological responses will be contaminated by previous trails (20, 22).

To cope with flaws in GNG and SS tasks, this study attempts to apply a two-choice Oddball (TCO) paradigm in the understanding of inhibitory control (20, 21, 23–25). In a TCO task, participants are required to response to both standard and deviant stimuli, so that the results are not contaminated by motor-response-related processes. Consequently, the TCO paradigm is thought to have a higher ecological validity in the evaluation of inhibitory control than others (e.g., standard GNG and SS tasks) (21, 23–25). TCO paradigms have been widely used in various applications, such as addiction (24), depression (25), restrained eaters (26), acute stress (27), visually induced motion sickness (28), avoidant attachment-style individuals (29), nonsuicidal self-injury (30), and male smokers (20). However, it has not fully investigated the issue of how to apply TCO paradigms to understand inhibitory control deficits in the comorbidity of ADHD and SLD.

Functional near-infrared spectroscopy (fNIRS) is a non-invasive method to measure brain activity by measuring the absorption of the near-infrared light between 650 and 950 nm through the intact skull (31–36). It is a relatively non-invasive and inexpensive functional neuroimaging technique that provides direct and quantitative measurement of cortical hemodynamic responses to cognitive tasks (31–34). Due to its easy applicability, high ecological validity, resistance to motion artifacts, and cost-effectiveness, fNIRS have advantages in some applications: (1) It can be applied to evaluate cortical hemodynamics in circumstances where other methods fail (e.g., during real-life social interaction or whole body movements) (36); (2) It is particularly suitable for children who may be afraid of tight surroundings (e.g., in MRI/PET scanners) or show motor restlessness (e.g., in ADHD) that interferes with motion-sensitive imaging methods such as MRI, EEG, MEG or PET (31–34); and (3) it allows for frequent measurement repetitions and can be easily used for longitudinal studies that become more and more important for the investigation of the development and treatment of psychiatric disorders (36).

Due to its advantages above, fNIRS has created new opportunities for investigating the neural and hyperscanning processes in various neurodevelopmental conditions, including ADHD or SLD (32–38). In particular, the majority of fNIRS studies focused on alterations in prefrontal cortex (PFC) activity during different experimental paradigms involving executive functions, such as Stroop tasks (39–41), WM tasks (42), the trail making test (43) or Go/NoGo paradigms (41, 44). Previous fNIRS studies have brought some insight into the understanding of aberrant cortical (e.g., prefrontal) hemodynamic responses in ADHD and SLD (32–34). However, as far as we know, there is no study in the literature attempting to investigate the comorbidity of SLD and ADHD by using fNIRS (especially with multiple channels).

Taken together, this study sought to reveal the differences between inhibition control features of children with ADHD, children with the comorbidity of ADHD and SLD), and healthy children in a multi-modal fashion (i.e., behavior plus fNIRS brain imaging). For this purpose, a total number of 90 children (aged 6-12 years) were recruited, including 30 ADHD (children with ADHD but without SLD), 30 ADHD+SLD (children with the comorbidity of ADHD and SLD), and 30 typically developing (TD) children. A 44-channel fNIRS equipment was adopted to measure behavioral and cortical hemodynamic responses for each participant during a TCO task. This study attempted to compare the differences in behavioral responses and brain activation of 44 channels among TD, ADHD, and ADHD+SLD groups. More particularly, this study hypothesized that inhibition control abilities (measured by behavioral responses and brain activation) would be further decreased in the ADHD children due to the comorbidity of SLD. To test this hypothesis became the main motivation of the current study. The associations between behavioral responses and brain activation were discussed as well.

## 2 Methods

The Ethics Committee of Nanjing Maternal and Child Health Hospital gave its approval to all study protocols and research techniques, ensuring that they adhered to the World Medical Association’s Declaration of Helsinki regarding the use of humans in testing. All participating children’s parents gave their informed consent, and each participant gave their oral consent before the experiment began.

### 2.1 Study design and participants

All participating ADHD and ADHD+SLD children (aged 6-12 years) were recruited from clinical cases in the Nanjing Maternal and Child Health Hospital from 2021 to 2022. This study also recruited TD children from a primary school in Nanjing. Hence, this study recruited three groups (i.e., TD, ADHD, and ADHD+SLD children). A senior expert (with professional experience more than 10 years) was naïve to group assignment and carried out the clinical measures and experimental data collection for all children within 5 days. The senior expert had training in administration of all tools used in this study.

Firstly, the senior expert conducted the IQ tests for each child using the Chinese version of the Wechsler intelligence scale for children (C-WISC). Children with IQ ≥ 85 were invited to participate the current study. Secondly, the senior expert conducted the diagnosis of ADHD for a child according to DSM-5 criteria and the Vanderbilt ADHD scales (45, 46) for teacher and parents based on his/her behavior in the last 6 months. Thirdly, for those diagnosed as ADHD, the senior expert further carried out the diagnosis of SLD as follows: (1) Academic achievements (including reading fluency and comprehension, mathematical calculations and reasoning, and writing fluency and accuracy) were within the bottom 10% of all children in the same grade; (2) Total score of the Preschool Learning Skills Scale (PLSS) was within the bottom 10% of all children in the same grade (47); and (3) Total score of the Learning Disability Screening Scale (PRS) was below than 65 (48).

The exclusion criteria included the following conditions: (a) receiving ADHD intervention (including psychotherapy and medication); (b) non-native Chinese speakers; (c) left handedness; (d) girls; (e) major physical diseases; (f) organic diseases of the nervous system; (g) intellectual disability, epilepsy, autism spectrum disorders and other severe neurodevelopmental disorders; and (h) incomplete clinical data related to the processing of evaluation.

Based on inclusion and exclusion criteria above, this study recruited 90 children to attend the experiments, including 30 TD children (age: 8.47 ± 1.67; IQ: 108 ± 10.76), 30 ADHD (age: 8.39 ± 1.38; IQ: 106 ± 12.17), and 30 ADHD+SLD (age: 8.13 ± 1.29; IQ: 104 ± 11.57). **Table 1** summarized the detailed demographic characteristics of participants. It is clear from **Table 1** that there was no significant difference of age (*F*=0.451, *p*=0.638) and IQ score (*F*=1.339, *p*=0.268) among the three groups (i.e., TD, ADHD, and ADHD+SLD groups). In addition, children in the TD group were matched with both the ADHD and ADHD+SLD groups in terms of age (*F*=0.451, *p*=0.638>0.05) and IQ (*F*=1.339, *p*=0.268>0.05).

**Table 1:** Demographic information of participants.

	TD group (*n*=30)	ADHD group (*n*=30)	ADHD+SLD group (*n*=30)	*F*	*df1/df2*	*p*
**Age (years)**	8.47 ± 1.67	8.39 ± 1.38	8.13 ± 1.29	*F*=0.451	2/87	0.638
**IQ score**	108 ± 10.76	106 ± 12.17	104 ± 11.57	*F*=1.339	2/87	0.268

### 2.2 Experimental paradigm

This study adopted the children’s version of the TCO paradigm (45, 46). Participants were tested in a quiet room. During this task, two different types of stimulus pictures were presented in the center of a 23.5-inch monitor, where each stimulus was presented for 800 ms with a stimulus interval of 200 ms. Participants were required to follow the following rules: (i) When a cartoon tiger or lion (baseline trial) was displayed, the participant was asked to press the left button (red button); and (ii) When an elephant (Oddball trial) was displayed, the participant was asked to press the right button (blue button).

**Figure 1:**
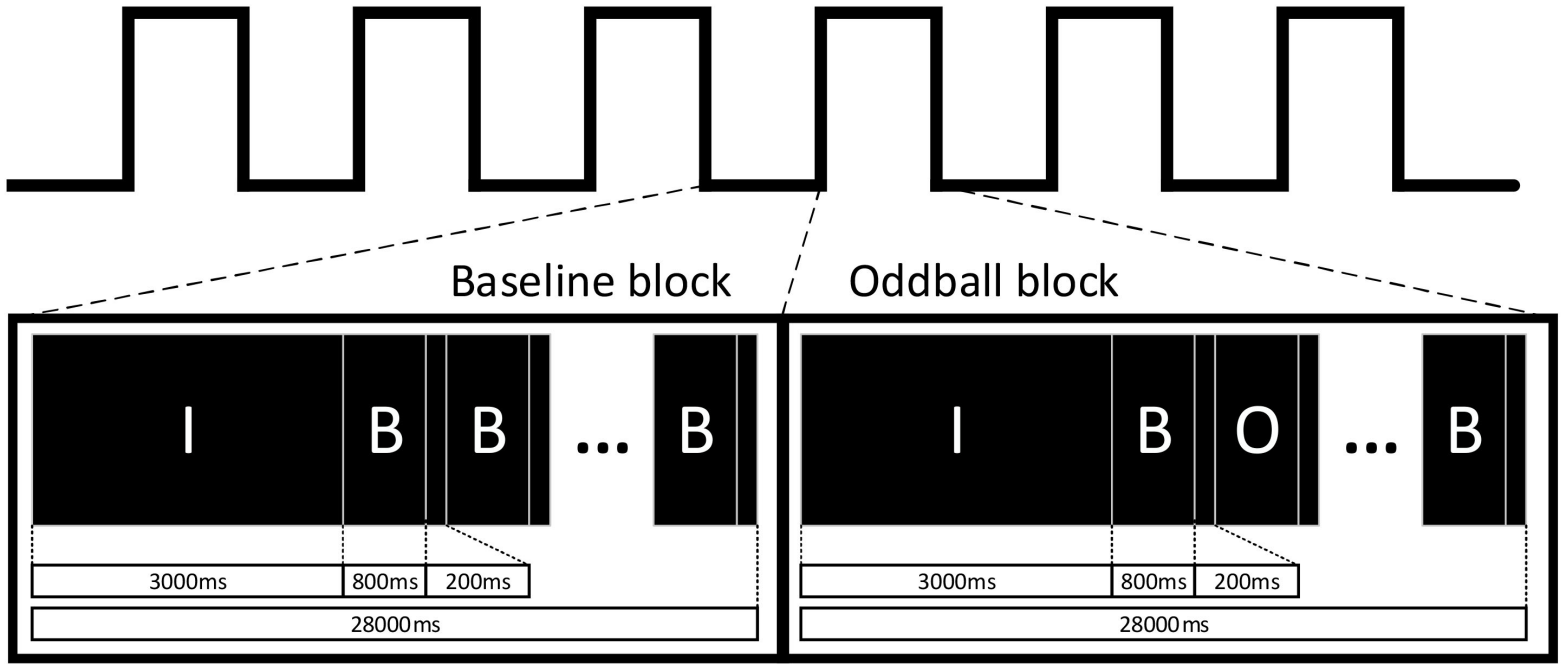
Experimental flow chart, including two kinds of blocks (i.e., Baseline and Oddball blocks), where “I” represents the prompt stage; “B” represents the baseline trials; and “O” represents the Oddball trials. Participating children were required to press the left or right button when different trials (i.e., baseline and Oddball trials) were presented.

As shown in **Figure 1**, the experimental task consisted of a total of 13 blocks. The first block was a Baseline block, while the following 12 blocks alternated six times between Baseline blocks and Oddball blocks. Each block included a 3-second instruction and a 24-second stimulus with a stimulus interval of 200 ms. During the Baseline block (all stimuli were baseline trials), participants were required to press the left button (red button). During the Oddball block, a random distribution of both stimuli appeared, with the ratio of baseline to oddball trials being 4:1. The experimental task lasted approximately 6 min. Before a formal experiment, participants were instructed to do some practice for the understanding of the whole experimental procedure.

### 2.3 Behavioral measures

As shown in **Figure 1**, there are two types of trails, i.e., baseline and Oddball trials, in the TCO task. For the *i*-th Oddball trial, the corresponding response time *OddballRT*(*i*) is defined as the time interval between the stimulus presentation and the button response. Similarly, *baselineRT*(*i*) and *totalRT*(*i*) are defined for the *i*-th baseline trials and all trails, respectively. Additionally, a state function *OddballS*(*i*) is defined, where *OddballS*(*i*)=1 if the *i*-th Oddball trial is correctly responded; and *OddballS*(*i*)=0 otherwise. Similarly, *baselineS* (*i*) and *totalS*(*i*) are defined for the *i*-th baseline trials and all trails, respectively.

Based on definitions above, three accuracy-related metrics, i.e., OddballACC, baselineACC, and totalACC, are computed as follow:


(1) $$\text{OddballACC}=(\frac{1}{NO})\sum_{i=1}^{NO}OddballS(i)$$

(2) $$\text{baselineACC}=(\frac{1}{Nb})\sum_{i=1}^{Nb}baselineS(i)$$

(3) $$\text{totalACC}=(\frac{1}{N})\sum_{i=1}^{Nt}totalS(i)$$

where *NO*, *Nb*, and *Nt* are the total number of Oddball trail, baseline trails, and all trials, respectively.

In addition, three response-time-related metrics, i.e., OddballRT, baselineRT, and totalRT, are computed as follows:


(4) $$\text{OddballRT}=\sum_{i=1}^{NO}\text{OddballRT}(i)*\text{OddballS}(i)/\sum_{i=1}^{NO}\text{OddballS}(i)$$

(5) $$\text{baselineRT}=\sum_{i=1}^{Nb}baselineRT(i)*\;baselineS(i)/\sum_{i=1}^{Nb}baselineS(i)$$

(6) $$\text{totalRT}=\sum_{i=1}^{Nt}totalRT(i)*totalS(i)/\sum_{i=1}^{Nt}TotalS(i)$$

where *NO*, *Nb*, and *Nt* are the total number of Oddball trail, baseline trails, and all trials, respectively.

Hence, six behavioral metrics (i.e., OddballACC, baselineACC, totalACC, OddballRT, baselineRT, and totalRT) can be obtained in the current study.

### 2.4 Acquisition and analysis of fNIRS data

During the TCO task, the oxy-hemoglobin (HbO) and deoxy-hemoglobin (HbR) concentration changes at the wavelengths of 695 and 830 nm were recorded using an fNIRS equipment (ETG-4100, Hitachi Medical Corporation, Japan), with sampling frequency 10Hz. This fNIRS equipment had 16 light sources and 14 detectors, evenly distributed over the left and right prefrontal regions according to the 10-10 transcranial positioning system. Hence, this fNIRS equipment actually had 44 neural channels covering bilateral superior frontal gyrus, middle frontal gyrus, and inferior frontal gyrus, angular gyrus, precentral gyrus, postcentral gyrus, supramarginal gyrus, superior temporal gyrus, and middle temporal gyrus (see **Table 2**).

**Table 2:** Estimated locations of 44 channels.

Channel	MNI	Anatomical label
CH01	(-28, 26, 58)	L superior frontal gyrus
CH02	(-45, 5, 58)	L middle frontal gyrus
CH03	(-56, -24, 57)	L postcentral gyrus
CH04	(-58, -50, 51)	L angular gyrus
CH05	(-26, 43, 46)	L middle frontal gyrus
CH06	(-45, 21, 51)	L middle frontal gyrus
CH07	(-59, -9, 46)	L postcentral gyrus
CH08	(-65, -36, 43)	L supramarginal gyrus
CH09	(-60, -62, 33)	L angular gyrus
CH10	(-44, 37, 37)	L middle frontal gyrus
CH11	(-59, 9, 36)	L precentral gyrus
CH12	(-67, -20, 32)	L supramarginal gyrus
CH13	(-67, -48, 23)	L supramarginal gyrus
CH14	(-40, 54, 24)	L middle frontal gyrus
CH15	(-56, 28, 23)	L inferior frontal gyrus
CH16	(-66, -3, 20)	L postcentral gyrus
CH17	(-70, -33, 9)	L superior temporal gyrus
CH18	(-65, -59, 0)	L middle temporal gyrus
CH19	(-51, 44, 8)	L inferior frontal gyrus
CH20	(-60, 15, 9)	L inferior frontal gyrus
CH21	(-70, -17, -5)	L middle temporal gyrus
CH22	(-69, -43, -11)	L middle temporal gyrus
CH23	(63, -44, 48)	R angular gyrus
CH24	(62, -18, 50)	R supramarginal gyrus
CH25	(50, 12, 52)	R middle frontal gyrus
CH26	(35, 32, 52)	R middle frontal gyrus
CH27	(65, -54, 29)	R angular gyrus
CH28	(70, -29, 36)	R supramarginal gyrus
CH29	(64, 0, 39)	R postcentral gyrus
CH30	(50, 27, 42)	R middle frontal gyrus
CH31	(32, 47, 42)	R middle frontal gyrus
CH32	(71, -38, 16)	R superior temporal gyrus
CH33	(70, -11, 24)	R postcentral gyrus
CH34	(62, 16, 26)	R precentral gyrus
CH35	(49, 41, 30)	R middle frontal gyrus
CH36	(69, -51, -6)	R middle temporal gyrus
CH37	(73, -26, 2)	R middle temporal gyrus
CH38	(67, 3, 14)	R postcentral gyrus
CH39	(58, 34, 16)	R inferior frontal gyrus
CH40	(44, 56, 19)	R middle frontal gyrus
CH41	(71, -38, -17)	R middle temporal gyrus
CH42	(71, -11, -11)	R middle temporal gyrus
CH43	(59, 22, 2)	R inferior frontal gyrus
CH44	(53, 47, 2)	R inferior frontal gyrus

The fNIRS data were preprocessed by the NIRS-KIT toolbox (47). The preprocessing of fNIRS data basically included the following four steps. First, we converted the raw intensity data into hemoglobin concentration units. Second, we removed linear drifts and kinetic artifacts in hemoglobin concentration data by a regression model and correlation-based method (48), respectively. Third, we removed high- and low-frequency noise by a bandpass filter with cutoff frequencies at 0.01-0.1 Hz. Fourth and finally, we calculated hemoglobin concentration variations by a modified Beer-Lambert law (49).

This study considered the HbO concentration only, due to its high sensitivity to changes in regional cerebral blood flow (50). The General Linear Model (GLM) has been widely used as a standard method for analyzing fNIRS data for channel activation analysis (51, 52). After completing the data preprocessing, this study used the GLM to determine whether a channel significantly activated or did not (53).

In order to extract significant activation channels, this study analyzed the HbO data of 44 channels using GLM for each participant from the three groups (i.e., TD, ADHD, ADHD+SLD groups), where the activation level of each channel was evaluated by the beta value of GLM.

### 2.5 Statistical analysis

To test the differences of inhibition control features among the three groups (i.e., TD, ADHD, and ADHD+SLD groups), this study conducted statistical analyses for 50 variables, including six behavioral metrics (computed according to Equations 1–6), as well as 44 beta values of GLM in 44 fNIRS channels. After confirming that our data passed the normality test and variance homogeneity test, we conducted a number of parametric ANOVA tests for comparisons of group difference, as well as *t*-tests for *post-hoc* multiple comparisons. Additionally, for *post-hoc* multiple comparisons, we utilized the Bonferroni correction to *p*-values to control the false discovery rate (FDR).

We also used the Pearson’s correlation analysis (with a Bonferroni correction) to test the relationship between the brain activation levels and behavioral measures.

All statistical analysis above was conducted with R language (version 4.0.2) and the significance level α was set at 0.05. The size of the effect was classified as a small effect (*η* ^2^ = 0.01; *d*=0.20), a medium effect (*η* ^2^ = 0.06; *d*=0.50), and a large effect (*η* ^2^ = 0.14; *d*=0.80). In particular, R package “ bruceR” was used in this study.

## 3 Results

### 3.1 Behavioral measures

This study conducted ANOVA analysis for six behavioral metrics (i.e., OddballACC, baselineACC, totalACC, OddballRT, baselineRT, and totalRT) to reveal the differences among the three groups (i.e., TD, ADHD, and ADHD+SLD groups). **Tables 3**, **4** summarized our results and showed that there were significant differences in OddballACC (*F*=15.117, *p*<0.001), baselineRT (*F*=4.249, *p*=0.017) and totalACC (*F*=6.209, *p*=0.003) among the three groups, but were not in OddballRT, baselineACC, and totalRT (*F*=1.214-2.259, *p*’s>0.183).

**Table 3:** ANOVA results for two behavioral measures.

Variables	TD group	ADHD group	ADHD+SLD group	ANOVA			
	M ± SD	M ± SD	M ± SD	*F*	*df1/df2*	*p*	*η* ^2^
**OddballACC**	85.8% ± 10.0%	74.9% ± 19.3%	61.9% ± 19.4%	15.117	2/87	<0.001^***^	0.258
**OddballRT(s)**	0.677 ± 0.083	0.647 ± 0.143	0.628 ± 0.130	1.214	2/87	0.302	0.027
**baselineACC**	92.1% 2.8.1%	88.1% 8.13.0%	86.6% 6.8.9%	2.259	2/87	0.111	0.049
**baselineRT(s)**	0.579.50.080	0.620.60.057	0.625.60.059	4.249	2/87	0.017^*^	0.089
**totalACC**	90.8% 0.7.1%	85.5% 5.12.3%	81.7% 1.10.1%	6.209	2/87	0.003^**^	0.125
**totalRT(s)**	0.599.50.075	0.625.60.058	0.636.60.058	1.733	2/87	0.183	0.038

*p<0.05; **p<0.01; ***p<0.001.

**Table 4:** *Post-hoc* multiple comparisons for OddballACC, BallRT and TotalACC.

Variables	ADHD+SLD vs. TD			ADHD vs. TD			ADHD+SLD vs. ADHD		
	*T*	*p^#^*	*d*	*t*	*p^#^*	*d*	*t*	*p^#^*	*d*
**OddballACC**	5.491	<.001^***^	1.418	2.503	0.043*	0.646	2.988	0.011^*^	0.772
**baselineRT**	2.658	0.028^*^	0.686	2.365	0.061	0.611	0.293	1.000	0.076
**totalACC**	3.509	0.002^**^	0.906	2.040	0.133	0.527	1.468	0.437	0.379

^#^represents p-value adjusted with Bonferroni correction; *p<0.05; **p<0.01; ***p<0.001.

Additionally, as shown in **Table 4**, multiple comparisons with a Bonferroni correction verified that: (1) both the ADHD and TD groups exhibited a higher value in OddballACC than the ADHD+SLD group (*p*’s<0.05, adjusted); (2) the TD group showed a higher value in OddballACC than the ADHD group (*p*<0.05, adjusted); (3) the ADHD+SLD group exhibited lower value in totalACC but higher value in baselineRT than the TD group (*p*’s ≤ 0.028, adjusted); and (4) there were no significant differences in totalACC and baselineRT between the ADHD and TD groups (*p*’s>0.05, adjusted), as well as between the ADHD and ADHD+SLD groups (*p*’s>0.05, adjusted).

### 3.2 Brain activation

**Figure 2:**
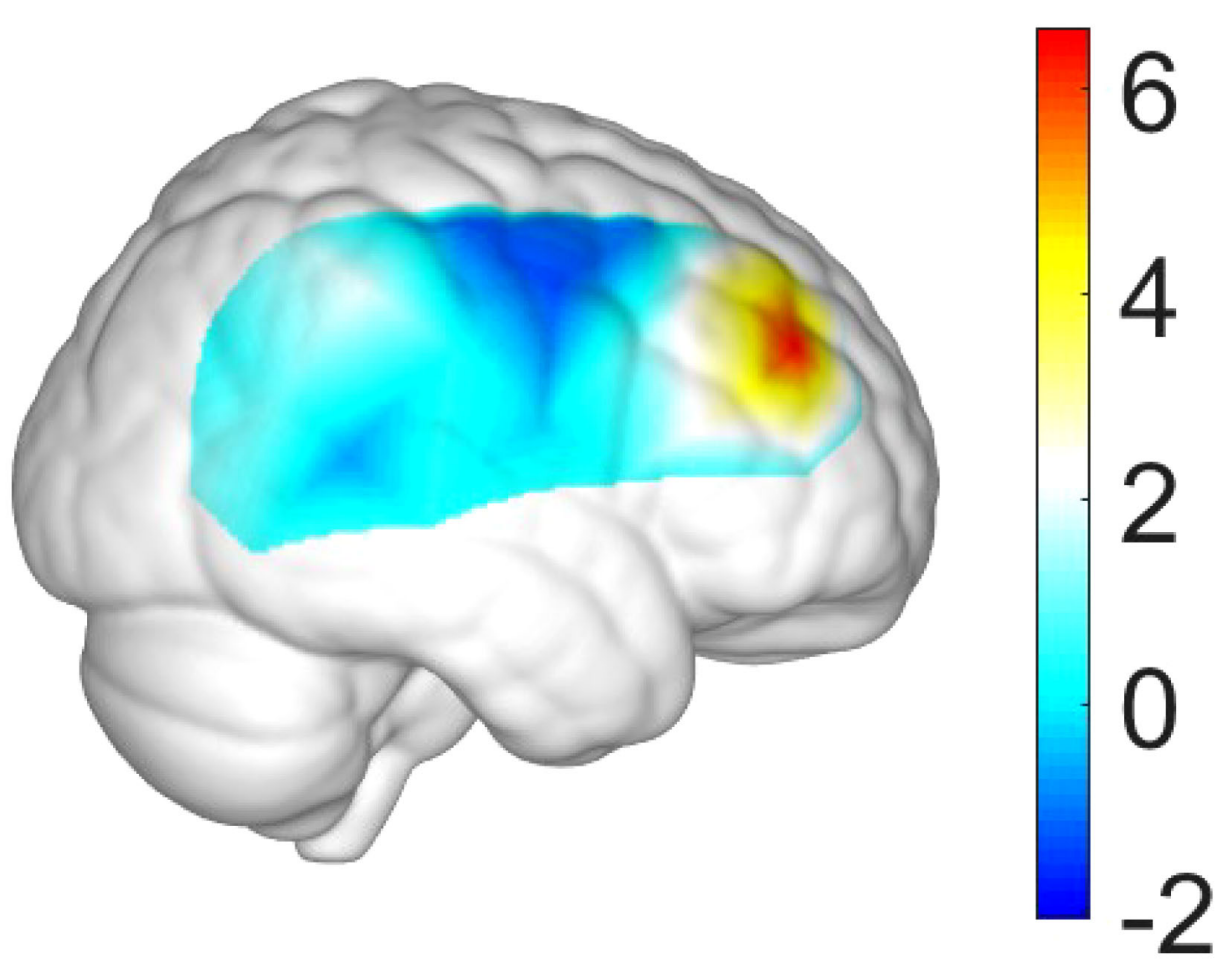
Activation map (i.e., map of *t* values) in the right hemisphere for the TD group.

This study conducted a number of *t*-tests for the beta values of GLM for each channel in the TD group. **Table 5**; **Figure 2** summarized our results, and showed that only three channels (i.e., CH26, CH30, and CH35) significantly activated in the TD group (*p’s*<0.05). The three channels located in the right middle frontal gyrus (MFG), or the border between the right inferior frontal gyrus (IFG) and the right MFG.

**Table 5:** Activated channels in the TD group.

Beta values	M ± SD	*t*	*p*
**CH26**	0.150 ± 0.232	3.537	0.020 *
**CH30**	0.166 ± 0.257	3.539	0.020 *
**CH35**	0.179 ± 0.145	6.594	<0.001 ***

*p<0.05; ***p<0.001.

**Figure 3:**
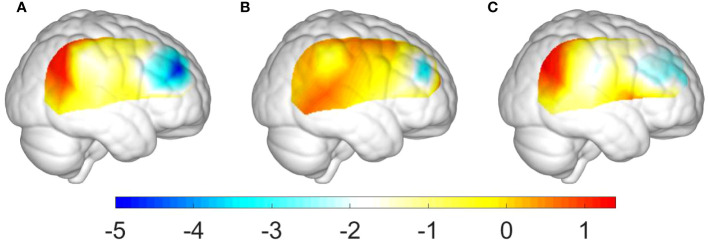
Group differences of activation levels in the right hemisphere: **(A)** between the ADHD+SLD and TD groups; **(B)** between the ADHD and TD groups; and **(C)** between the ADHD+SLD and ADHD groups.

This study conducted ANOVA analysis for the beta values in channels CH26, CH30, and CH35 to reveal the differences among the three groups (i.e., ADHD, ADHD+SLD, and TD groups). **Table 6** and **Figure 3** summarized our results and showed that there were significant differences in all variables (i.e., the beta values in channels CH26, CH30, and CH35) among the three groups (*p’s*<0.01).

**Table 6:** ANOVA results for beta values in three channels.

Beta values in different channels	TD Group	ADHD group	ADHD+SLD group	ANOVA			
	M ± SD	M ± SD	M ± SD	*F*	*df1/df2*	*p*	*η* ^2^
**CH26**	0.150 ± 0.232	0.097 ± 0.203	0.067 ± 0.360	5.109	2/87	0.008^**^	0.105
**CH30**	0.166 ± 0.257	0.097 ± 0.194	0.042 ± 0.307	5.098	2/87	0.008^**^	0.105
**CH35**	0.179 ± 0.145	0.051 ± 0.110	0.075 ± 0.235	16.284	2/87	<0.001^***^	0.272

**p<0.01; ***p<0.001.

Additionally, as shown in **Table 7**, multiple comparisons with a Bonferroni correction verified that: (i) the TD group exhibited a higher activation level in channels CH26, CH30, and CH35 than the ADHD+SLD group (*p*’s<0.01, adjusted); (ii) the TD group exhibited a higher activation level in channel CH35 than the ADHD group (*p*’s<0.05, adjusted), but did not in channels CH26 and CH30; and (iii) the ADHD group exhibited a higher activation level in channel CH35 than the ADHD+SLD group (*p*’s<0.05, adjusted), but did not in channels CH26 and CH30.

**Table 7:** *Post-hoc* multiple comparisons for beta values in channels CH20, CH26, and CH35.

Beta values in different channels	ADHD+SLD vs. TD			ADHD vs. TD			ADHD+SLD vs. ADHD		
	*t*	*p^#^*	*d*	*t*	*p^#^*	*d*	*t*	*p^#^*	*d*
**CH26**	3.064	0.009^**^	0.791	0.743	1.000	0.192	2.321	0.068	0.599
**CH30**	3.135	0.007^**^	0.809	1.041	0.903	0.269	2.094	0.117	0.541
**CH35**	5.707	<0.001^***^	1.473	2.874	0.015^*^	0.742	2.833	0.017^*^	0.731

^#^represents p-value adjusted with Bonferroni correction; *p<0.05; **p<0.01; ***p<0.001.

### 3.3 Correlation analysis

**Figure 4:**
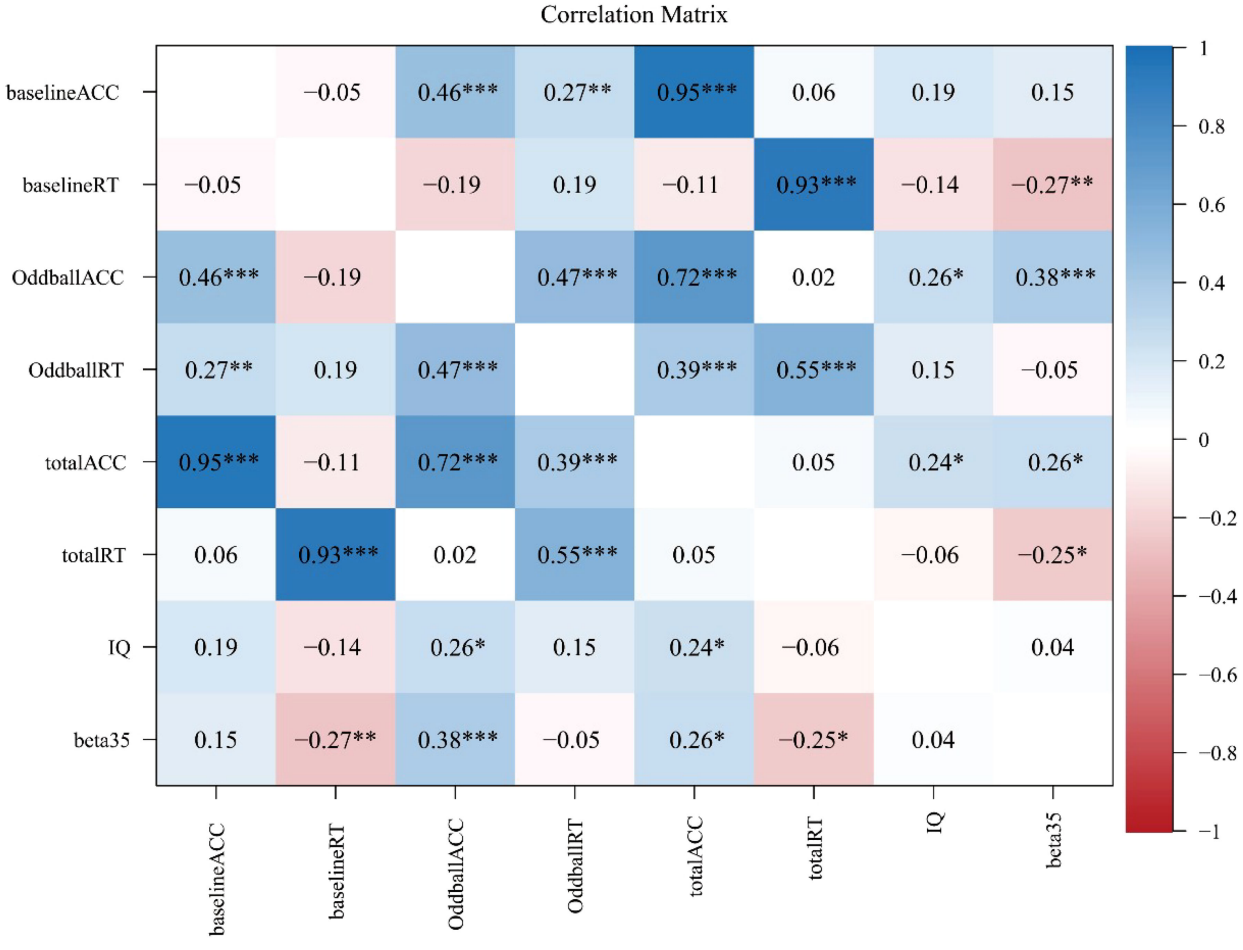
Correlation analysis results with a Bonferroni correction, where *, **, and *** represent *p*<0.05, *p*<0.01, and *p*<0.001, respectively.

It is interesting to test the correlation among the activation level of channel CH35 (evaluated by beta35, the beta value in CH35), IQ score, and six behavioral metrics (i.e., OddballACC, baselineACC, totalACC, OddballRT, baselineRT, and totalRT) by Person’s correlation method with a Bonferroni correction. **Figure 4** summarized our results.

As shown in **Figure 4**, some significant results included that: (1) beta35 was significantly correlated with baselineRT (*r*=-0.27; *p*<0.01, adjusted), OddballACC (*r*=0.38; *p*<0.001, adjusted), totalACC (*r*=0.26; *p*<0.05, adjusted), and totalRT (*r*=-0.25; *p*<0.05, adjusted), but was not with other variables (*p*’s>0.05, adjusted); (2) IQ score was significantly correlated with OddballACC (*r*=0.26; *p*<0.05, adjusted) and totalACC (*r*=0.24; *p*<0.05, adjusted), but was not with other variables (*p*’s>0.05, adjusted); (3) there was a significant correlation between OddballACC and OddballRT (*r*=0.47; *p*<0.001, adjusted), but was not between baselineACC and baselineRT and between totalACC and totalRT (*p*’s>0.05, adjusted); (4) there was a significant correlation between OddballACC and baselineACC (*r*=0.46; *p*<0.001, adjusted), between OddballACC and totalACC (*r*=0.72; *p*<0.001, adjusted), and between baselineACC and totalACC (*r*=0.95; *p*<0.001, adjusted); and (5) there was a significant correlation between OddballRT and totalRT (*r*=0.55; *p*<0.001, adjusted) and between baselineACC and totalACC (*r*=0.93; *p*<0.001, adjusted), but was not between OddballRT and baselineRT (*p*>0.05, adjusted).

## 4 Discussion

This study aimed to reveal the differences between inhibition control features of TD, and ADHD, and ADHD+SLD children in a multi-modal fashion. For this purpose, we adopted an fNIRS equipment to capture behavioral responses and 44 channels of cortical hemodynamic responses during a TCO task, and compared the behavioral response metrics and brain activation of 44 channels among the TD, ADHD, and ADHD+SLD children. Findings showed that the ADHD+SLD children exhibited decreased behavioral response accuracy and brain activation level in some brain channels (e.g., channel CH35) than both the ADHD and TD children. This implies that reduced inhibition control ability in the ADHD children due to coexisting SLD may be observed.

Findings of behavioral analysis (see **Tables 3**, **4**) showed that: (1) both the ADHD and TD groups exhibited a higher value in OddballACC than the ADHD+SLD group; and (2) the TD group showed a higher value in OddballACC than the ADHD group. This implies that the co-occurrence of SLD may further deteriorate the functional impairments of ADHD that they already have. In the meanwhile, there were no significant differences in other five behavioral metrics (i.e., baselineACC, totalACC, OddballRT, baselineRT, and totalRT) between the ADHD and ADHD+SLD groups. This indicates that OddballACC (i.e., the response accuracy in deviant stimuli) is the most sensitive metric in identifying the differences between the ADHD and ADHD+SLD children.

The TCO paradigm involves two stimuli (displaying in the same sensory channel), which are presented at random but with significantly different probabilities. The stimuli with high probability are called standard stimuli (called baseline trails here); whereas, the stimuli with low probability are called deviant stimuli (called Oddball trails here). In this task, participants are required to respond to both high-frequency stimuli (standard stimuli) and low-frequency stimuli (deviant stimuli) as accurately as possible, then press different keys to make responses as quickly as possible. The pressing frequency of standard stimuli (baseline trails) is much higher than that of deviant stimuli (Oddball trails). Therefore, when low-frequency deviant stimuli (Oddball trails) appear, participants need to suppress the established dominant pressing tendency toward standard stimuli (baseline trails) to ensure correct responses to deviant stimuli (21, 23–25). Due to this inhibition mechanism, the TCO paradigm is thought to have much higher ecological validity for the evaluation of inhibitory control than others (such as standard GNG and SS tasks).

From a neuroscience perspective, the TCO task is stimulus-driven and involves a bottom-up process. The identification of Oddball target stimuli may inspire the ventral frontal network, which is composed of the ventral prefrontal cortex and the area surrounding the temporal parietal junction (54). Findings showed that children with ADHD exhibited decreased activation levels in the ventral prefrontal cortex. This is consistent with previous studies showing that the activation of regions MFG and IFG was insufficient in children with ADHD who had not receive any medication treatment (46, 55). Additionally, our results illustrated that the activation levels of children with ADHD may be further decreased due to the comorbidity of SLD. This finding, in combination with behavioral response comparison results, indicates that decreased behavioral response accuracy and brain activation level may be applied to interpret the functional impairments in children with ADHD, as well as that the comorbidity of SLD could exacerbate the functional impairments of children with ADHD that they already have. Hence, this study has verified our hypothesis that the comorbidity of SLD may have an additive influence on functional impairments for children with ADHD.

IQ tests have been widely used to map the structure of neurodevelopmental disorders (including ADHD and SLD), as well as depict the cognitive strength and weaknesses of an individual child for intervention procedure (56–59). The relation between IQ and EF has been examined (56, 57) as well. A meta-analysis showed that compared to healthy controls, children with ADHD have lower overall intellectual abilities (58). Some studies even found that the co-occurrence of ADHD and low IQ has genetic origins (59), and children who have ADHD and low IQ have poorer prognosis than those with ADHD alone (60). In addition, IQ levels are important predictors of outcome, even in children with ADHD and IQ in the normal range (60). Some studies have been explored cognitive profiles of children with ADHD and SLD using IQ tests (61, 62). For instance, a recent study (63) using WISC-V showed that compared to children with SLD, children with ADHD+SLD did not show specific impairments in any particular cognitive domain but rather non-specific impairment in almost all indices. Hence, even though there is no group difference IQ is not a covariate in the current study, it is still interesting to test whether IQ may affect behavioral and neural features in the ADHD and ADHD+SLD children.

On the other hand, many fNIRS studies did not consider the influence of IQ score on the difference of behavioral and brain-functional responses for both ADHD and TD children, and thus did not match the IQ score for both groups. Some research (45, 46) insightfully discussed the effect of IQ on brain activation, but the results did not display significant correlation between both ADHD and TD groups. However, our findings (see **Figure 3**) found that IQ score had a significant correlation with response accuracy (e.g., OddballACC and totalACC).

Strengths of the current study include the multi-modal analysis, the use of TCO paradigm (with higher ecological validity than GNG and SS tasks), the use of fNIRS with multiple channels, and positive results of this study. However, there are also some limitations. Firstly, we examined the differences in behavioral and cortical hemodynamic responses among the TD, ADHD, and ADHD+SLD chidlren. However, the conclusions in the study were constrained to some extent since we did not take into account a group solely consisting of children with SLD. The inclusion of the SLD group would allow for a deeper comparison among the TD, ADHD, SLD, and ADHD+SLD groups, shedding light on whether there are any differences due to the comorbidity or SLD alone. Secondly, we exclusively recruited boys to participate in our study. It is unclear whether the inclusion of girls in the sample would have an impact on the current study’s findings. Thirdly and finally, we employ the TCO paradigm to understand inhibitory control characteristics, instead of the widely used GNG and SS tasks. It deserves to compare the behavioral and cortical hemodynamic responses when different tasks are adopted.

Given the strengths of the current study in terms of the multi-modal fashion and significant group differences in OddballACC among the TD, ADHD, and ADHD+SLD children, the multi-modal technique (with multiple-channel fNIRS device) might be applied to reveal decreased inhibition control abilities in the ADHD children due to the comorbidity of SLD.

## Data availability statement

The original contributions presented in the study are included in the article/supplementary material. Further inquiries can be directed to the corresponding author.

## Ethics statement

The studies involving humans were approved by The Ethics Committee of Nanjing Maternal and Child Health Hospital. The studies were conducted in accordance with the local legislation and institutional requirements. Written informed consent for participation in this study was provided by the participants’ legal guardians/next of kin.

## Author contributions

FL: Writing – original draft, Visualization, Software, Methodology, Investigation, Formal Analysis, Data curation, Conceptualization. XC: Validation, Supervision, Resources, Funding acquisition, Writing – original draft, Visualization, Methodology, Investigation, Data curation, Conceptualization. DY: Writing – review & editing, Software, Project administration, Formal Analysis, Writing – original draft, Visualization, Validation, Supervision, Resources, Methodology, Investigation, Funding acquisition, Data curation, Conceptualization.
